# Broadscale reconnaissance of coral reefs from citizen science and deep learning

**DOI:** 10.1007/s10661-025-14261-6

**Published:** 2025-06-27

**Authors:** Christopher L. Lawson, Kathryn M. Chartrand, Chris M. Roelfsema, Aruna Kolluru, Peter J. Mumby

**Affiliations:** 1 Marine Spatial Ecology Laboratory, Centre for Conservation and Biodiversity Science, School of the Environment, The University of Queensland, Brisbane, Australia; 2 Centre for Tropical Water and Aquatic Ecosystem Research, James Cook University, Cairns, Australia; 3 Marine Ecosystems Monitoring Lab, School of the Environment, The University of Queensland, Brisbane, Australia; 4 Dell Technologies Inc, Round Rock, TX USA

**Keywords:** Benthic cover, Large-scale data, Volunteer monitoring, Coral classification, Image analysis, Resource allocation

## Abstract

Coral reef managers require various forms of data. While monitoring is typically the preserve of scientists, there is an increasing need to collect larger scale, up-to-date data to prioritise limited conservation resources. Citizen science combined with novel technology may achieve data collection at the required scale, but the accuracy and feasibility of new tools must be assessed. Here, we show that a citizen science program that collects large field-of-view benthic images and analyses them using a combination of deep learning and online citizen scientists can produce accurate benthic cover estimates of key coral groups. The deep learning and citizen scientist analysis methods had different but complementary strengths depending on coral category. When the best performing analysis method was used for each category in all images, mean estimates from 8086 images of percent benthic cover of branching *Acropora*, plating *Acropora*, and massive-form coral were ~ 99% accurate compared to expert assessment, and > 95% accurate at all coral cover ranges tested. Site-level accuracy of 95% was attainable with 18–80 images. Power analyses showed that up to 114 images per site were needed to detect a 10% absolute difference in coral cover per category (power = 0.8). However, estimates of ‘all other coral’ as a single category achieved 95% accuracy at only 60% of sites and for images with 10–30% coral cover. Overall, emerging technology and citizen science present an attainable tool for collecting inexpensive, widespread data that can complement higher resolution survey programs or be an accessible tool for locations with limited scientific or conservation resources.

**Supplementary Information:**

The online version contains supplementary material available at 10.1007/s10661-025-14261-6.

## Introduction

Ecosystem management needs various forms of data (Grêt-Regamey et al., 2017; Lindenmayer et al., 2008). Since the 1960 s, coral reef researchers have employed methods ranging from detailed, 20 m line-intercept transects (Loya, 1972) to aerial assessments covering hundreds of kilometres (Purkis, 2018). Line transect and quadrat sampling provide high taxonomic resolution and statistical rigor (English et al., 1997), but they are resource-intensive and spatially limited. Conversely, techniques such as aerial surveys and satellite remote sensing can cover extensive areas but lack detail to differentiate even broad groups of photosynthetic organisms (Hedley et al., 2016). While towed-diver surveys enable rapid in-water assessments, they rely on real-time observations that can be subject to observer bias without careful training and quality control, which inherently limits their scalability (Miller & Müller, 1999). Video transects can offer species-specific detail and replicability, but require extensive data storage, processing and in-water support for divers (Carleton & Done, 1995; Mallet & Pelletier, 2014). The evolution and combination of these monitoring approaches has proven essential, as together they have provided evidence for conservation policies (Hughes et al., 2017), revealed patterns of ecological change (De’Ath et al., 2012), and helped identify areas requiring immediate intervention (Knowlton & Jackson, 2008). While many government and research programs continue to conduct long-term reef monitoring focused on accurate estimates at high taxonomic resolution (Edmunds, 2024; Reverter et al., 2022), there is a growing need to complement these with coarser, rapid, in-water reconnaissance over large areas (Edmunds & Bruno, 1996; Mumby et al., 2021). Such up-to-date broadscale reconnaissance will inform where to prioritise limited conservation resources in the face of unprecedented global disturbances (Reverter et al., 2022; Swinfield et al., 2024).

One method to achieve broadscale reconnaissance is citizen science, whereby effort is crowdsourced from distributed participants. Citizen science has contributed data on coral reefs for decades. In the 1980’s, Raleigh International conducted dedicated project-based expeditions and marine surveys by trained citizen scientists (Beames, 2004). In the 1990 s, Coral Cay Conservation trained citizen scientists to collect data in support of establishing coral reef management plans in Belize (Mumby et al., 1995). More recently, Reef Check engages trained citizen scientists to capture percent cover of 10 benthic cover categories using point intercept transects; it collects data that are ~ 93% accurate and aims to support science and management decisions (Done et al., 2017; Hodgson, 1999). Established in 2007, Reef Life Survey uses selectively chosen and trained citizen scientists to collect high-quality data on Scuba, supporting global science and conservation efforts (Edgar & Stuart-Smith, 2014).

There are also government-run citizen science programs such as Reef Health Impact Surveys and Eye on the Reef, operated by the Great Barrier Reef Marine Park Authority in Australia (Beeden et al., 2014). Reef Health Impact Surveys provide ‘advanced in-water training’ to citizen scientists to collect data in a structured program. The Eye on the Reef mobile application is simpler and relies on opportunistic sampling that enables observational data collection by anyone on the Great Barrier Reef.

The CoralWatch citizen science program was established in 2002, creating a simple tool to assess the presence of coral bleaching by comparing in situ coral colour with a calibrated coral health chart. CoralWatch differs from many previous programs because it does not require substantial training and enables anybody to collect data, resulting in a large, opportunistically collected database (Marshall et al., 2012). CoralWatch currently comprises 17% of all publicly accessible bleaching data globally through its online data portal (unpublished data, C. Roelfsema).

Some of the limitations for citizen scientists to participate in accurate data collection may be removed by using technology such as deep learning (McClure et al., 2020). Deep learning, a subdomain of artificial intelligence, is a computational approach in which systems learn patterns from data, rather than following explicit instructions, enabling them to solve tasks based on examples rather than pre-defined solutions (Mitchell, 1997). Deep learning has dramatically increased the efficiency of environmental image analysis (e.g. González-Rivero et al., 2020). However, current deep learning tools for coral reefs mostly rely on consistent, high-quality photographs of quadrats (Courtney et al., 2022; González-Rivero et al., 2020; Schürholz & Chennu, 2023). While such photographs could be taken by citizen scientists, it requires dedicated Scuba logistics, which best suits the capacity of professional scientists engaged in monitoring reef state. Opening image collection to citizen scientists without training, specialist equipment, and with flexible logistics including snorkelling, would vastly expand the scope of data collection.

The Great Reef Census is a citizen science project that started on the Great Barrier Reef, Australia. The Great Reef Census utilises two types of citizen scientist: those who collect underwater images in the field and those ‘virtual volunteers’ who help analyse the resulting images online. The latter group are based all over the world and do not need access to the reef. Indeed, many virtual volunteers engaged in the study did not have access to the reef due to distance, resources or physical limitations. For in-water field surveys, citizen scientist tourists and reef industry workers capture images without specialised equipment or formal training. The only training required is reading a simple 2-page methods protocol. These images are then analysed using deep learning and by online citizen scientists to estimate benthic cover. A key question is if using deep learning reduces the barrier to entry for non-experts to participate in basic image analysis. Deep learning is generally faster at recognising shapes and is rapidly improving, but human vision may still outperform when complexities are introduced such as texture, shadows or poor water visibility (Rubbens et al., 2023).

There is a need to assess if citizen science-based benthic photo analysis can provide valid data to inform management, restoration or science. If image collection can be achieved by nearly anyone and analysis can be distributed to deep learning (artificial intelligence; hereafter ‘AI’) and citizen scientists globally, this would enable a vast expansion of the scope of data collection relative to traditional tools. However, achieving massive scaling of data collection requires a trade-off in precision, accuracy and taxonomic resolution. Because scale and accessibility for non-experts are limited by the complexity of species-level identification, here we do not identify species, which are constantly under revision and even beyond the skillset of many scientists (Ramírez-Portilla et al., 2022). Yet, measuring cover of select coral morphologies can still inform many management actions such as pest control and marine park planning, and morphological information by genus is important for key ecosystem functions like bioconstruction of reefs (Wolfe et al., 2020). Here, we focus on the capacity of citizen science to estimate cover of key coral morphologies that commonly dominate on the Great Barrier Reef: branching *Acropora*, plating *Acropora* and massive-form corals such as *Porites* or *Platygyra* (Veron, 2000). Branching and plating *Acropora* are fast-growing coral that are important for reef recovery following disturbance but are vulnerable to threats like crown-of-thorns starfish and cyclones (Loya et al., 2001; Ortiz et al., 2021; Pratchett et al., 2020). Massive corals are slower growing yet more resistant to threats and exhibit longevity that is important for sustaining reef accretion and persistence (Loya et al., 2001; Pratchett et al., 2020; Wolfe et al., 2020). Protecting populations of these coral groups can give outsized ecological benefit (Ortiz et al., 2021; Wolfe et al., 2020).

Our overall aim is to assess if large field-of-view benthic images of the reef collected by citizen scientists can provide sufficiently reliable information for reef management. To achieve this aim, our first objective is to assess if AI-alone or AI-supported citizen scientist analysis can accurately quantify the cover of three coral groups in benthic images collected by citizen scientists. Next, given the variability in accuracy among images, we ask how many images are needed to achieve a reasonable level of accuracy for a survey site, and how many online citizen scientists are needed to analyse each image. Finally, we run a series of power analyses to determine the number of images needed to also account for the natural heterogeneity of the reef.

## Methods

### Image collection and analysis

**Fig. 1:**
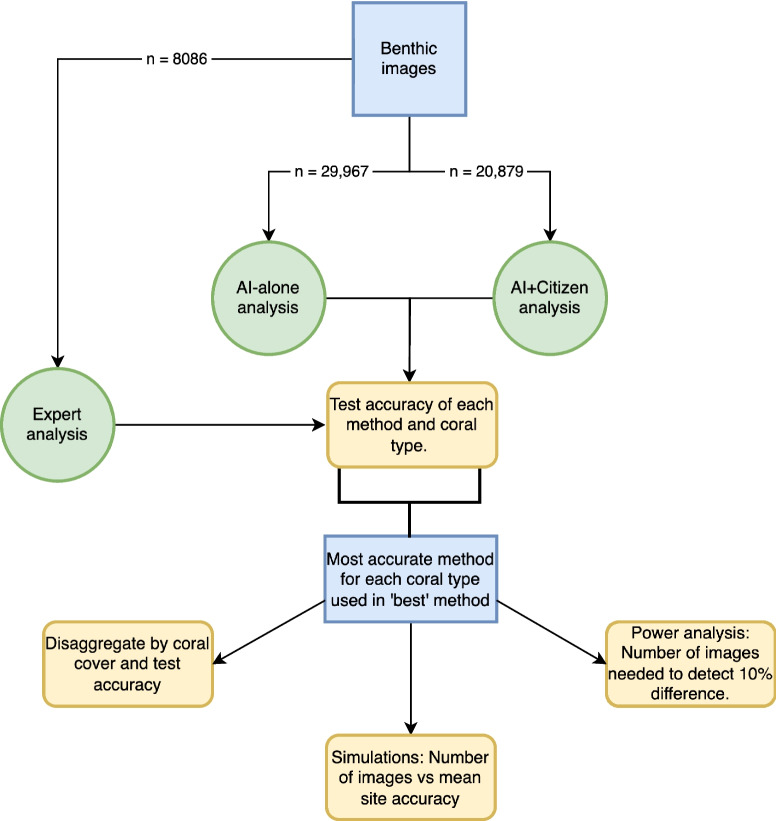
Summary flowchart of methods. Blue rectangles represent data, green circles represent methods of image analysis, and yellow rounded boxes represent statistical analyses of performance. Figure made with draw.io (JGraph, 2024)

We analysed benthic images collected by citizen scientists using three methods: a semantic segmentation deep learning model that generates and labels polygons of coral colonies (‘AI-alone’), an online analysis platform whereby citizen scientists label the polygons generated by the deep learning model (‘AI + Citizen’), and ‘expert’ analysis which was used to assess the performance of the other two methods. We used the results to explore if integrating deep learning into the workflow of citizen science analysis can improve the accuracy of derived coral cover estimates compared to using deep learning in isolation (Fig. 1).

### In-water survey methodology

Images (*n* = 29,967) were collected as part of the Great Reef Census from September 2022–February 2023 at 1512 sites distributed over 211 of the ~ 3000 reefs across the Great Barrier Reef, Australia. The Great Reef Census follows a simple survey methodology to collect large field-of-view benthic images. Volunteer citizen scientists (~ 70) were tasked with capturing random images of reef slopes or bommies at depths between 3 and 20 m. While participants could survey any reef, a priority map of reefs was provided to guide the most ‘valuable’ reefs to survey based on relevance to government managers, scientists or ecological importance, for example as a key source of larval dispersal (Mumby et al., 2021). Shallow reef tops (0–3 m) were excluded due to the difficulty of obtaining large field-of-view benthic images.

The survey protocol was designed to be easy, without the need for advanced training or scientific equipment. Images were collected on snorkel further from the substrate than standard photoquadrat surveys – i.e. 3–5 m compared to 1 m (Williams et al., 2019) – using basic handheld cameras such as GoPros (www.gopro.com). Images were captured parallel to the reef, with snorkelers duck-diving as required. Participants were told to capture images every 10 fin kicks, worked in pairs, and aimed to photograph reef sections approximately 5 m × 5 m in each image, with a minimum of 20 images per person per survey. Participants were instructed to survey at least three sites of a reef, separated by a minimum distance of 200 m. Preferably each site was located on a different aspect of the reef, i.e. north, south, east/windward or west/leeward, assuming safe and feasible logistics. Images were uploaded to the Great Reef Census web-based platform (www.greatreefcensus.org) with corresponding time and GPS coordinates. GPS coordinates were given for each image if a towed GPS unit was used, otherwise GPS coordinates were noted at the beginning of each survey from the mother vessel, the tender vessel or the camera’s internal GPS while it was above water.

### Expert validation data

To assess the accuracy of the AI-alone and AI + Citizen analyses, a subset of images were analysed with high accuracy using manual analysis by paid scientists skilled in coral identification and other benthic categories (hereafter referred to as ‘expert’ data).

**Fig. 2:**
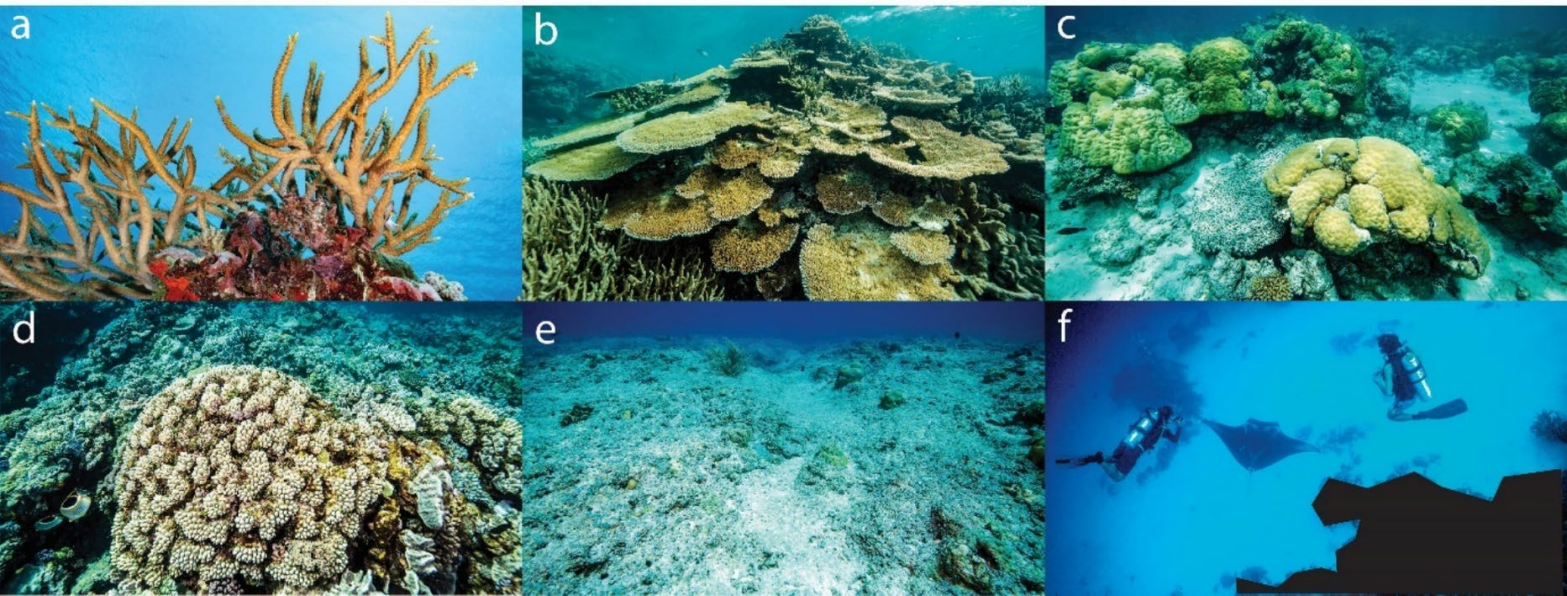
Category label options used for expert analysis, AI-alone analysis and the AI + Citizens online analysis platform. **a** ‘Branching coral’—branching coral of genus *Acropora*. **b** ‘Plating coral’—plating/table coral of genus *Acropora*. **c** ‘Massive coral’. **d** ‘Other coral’—all other coral types. **e** ‘Reef substrate’—any hard benthic surface suitable for coral growth. **f** ‘Water, sand and shadow’—any region not included in the other categories, consisting of the background water column, bare sand, shadow or other objects that preclude substrate identification. Photo credit: Peter J Mumby

To establish an efficient method of expert analysis, 615 images were first analysed by two methods: a ‘detailed’ method and a ‘visual’ method (Jokiel et al., 2015; Josephitis et al., 2012). The ‘detailed’ method used a custom-built software to draw polygons manually around individual coral colonies and assign a label corresponding to the categories of interest. The label options were branching *Acropora* (hereafter ‘branching’), plating *Acropora* (hereafter ‘plating’), massive-form coral (hereafter ‘massive’), all other coral (hereafter ‘Other’), ‘reef substrate’, ‘water, sand, and shadow’ and ‘I don’t know’ (Fig. 2). The total area of each coral category’s polygons in each image were then calculated. Coral categories were presented as percent of total colonisable reef substrate, i.e. excluding sand/water/shadow. The ‘visual’ method used a different custom-built software that placed a 9-cell grid (3 × 3) over each image. Each of the nine grid cells therefore comprised 11.1% of the total image. Experts visually assessed the proportion of each of the nine grid cells comprised of each coral category and assigned a percentage between 0 and 100. The percentage of each coral category in each grid cell (0–100) was multiplied by 0.111 (11.1%) to derive the cover in that grid cell as a percentage of the total image. Then, for each coral category, all grid cell values were summed to obtain the total cover of each coral type in each image. There was no significant difference in absolute coral cover between the ‘detailed’ and ‘visual’ methods (*p* = 0.6, mean difference =  − 1.5%, *n* = 615, Wilcoxon signed-rank test). As a result, we used the faster ‘visual’ method to maximise the number of images analysed. Using the ‘visual’ method, 8086 images were analysed by three experts. Images were randomly assigned to experts; if the same image was analysed by multiple experts, the average values for each coral cover category were taken.

### Deep learning model development (AI-alone analysis)

A semantic segmentation model (Guo et al., 2018) was trained to identify coral morphology in citizen science imagery. SegFormer-B5 was used to develop the segmentation model (Xie et al., 2021). SegFormer employs a hierarchical transformer backbone with a Multi-Level Perception (MLP) decoder that enables efficient semantic segmentation. As the largest SegFormer variant, the B5 architecture features a hierarchical transformer encoder that processes visual tokens at multiple scales (from 1/4 to 1/32), combined with a lightweight all-MLP decoder. This architecture allowed the model to capture fine-grained spatial features and contextual relationships within coral imagery. The B5 variant’s large parameter capacity and multi scale feature learning capabilities made it particularly suitable for analysing the variability in coral shapes, sizes and colours, as well as the complex underwater environment with challenging lighting conditions and diverse backgrounds. The model was implemented in Python using PyTorch and trained on a Dell Technologies HPC GPU-Accelerated System, utilising a Dell EMC PowerEdge server cluster (Table 1).

**Table 1:** Parameter values used in training the semantic segmentation (AI) model

Parameter	Value	Comments
Model architecture	SegFormer-B5	Largest SegFormer variant with ~ 84 M parameters
Encoder	Hierarchical transformer	4 stages with [3,6,40,3] transformer blocks
Decoder	All-MLP	Light weight decoder for efficient inference
Crop size	640 × 640 pixels	Provided a balance between computational efficiency and the preservation of crucial spatial features in the coral imagery
Batch size	7	Optimised memory usage on available hardware while ensuring stable gradient updates
Learning rate	0.00006	This relatively small learning rate was required for the fine-tuning process, enabling the model to gradually adjust to the intricacies of coral morphology without overshooting optimal parameter values
Learning rate schedule	Constant, with no additional scheduling mechanisms	This approach was chosen after observing that the model’s convergence was stable and that introducing a learning rate decay did not significantly improve performance during preliminary trials
Maximum epochs	30 epochs	Determined through iterative experiments to ensure that the model had sufficient opportunities to learn while avoiding overfitting
Early stopping patience value	20 epochs	Training would halt if no improvement in the validation mean Intersection over Union (IoU) score was observed over 20 consecutive epochs
Early stopping mode	Maximum	Ensured that the best-performing model was retained based on IoU maximisation

To train the segmentation model, 7505 large field-of-view benthic images collected by citizen scientists as part of the Great Reef Census expeditions (2020–2021) were annotated using the ‘detailed’ expert analysis method described earlier. A custom-built software was used to delineate key coral morphologies digitally and assign labels to each polygon, based on the same predefined categories used in expert analysis. These labelled polygons were converted into JSON files, which served as input for segmentation model training (Table 1). The dataset was split into an 80:20 ratio: 6004 images were used as the training set, and 1501 images were used as the validation set. The validation set was used during training to monitor the model’s performance and adjust its parameters to learn effectively (Table 1).

After training, the model was applied to 29,967 images that were not used during the training phase, generating segmentation masks by assigning each pixel to one of the predefined categories. For each image, the total number of pixels classified into each category was divided by the image’s known total pixel count to calculate the percent cover of each category. The cover of the coral categories were presented as percent of total colonisable reef substrate, i.e. excluding sand/water/shadow. These percent coral cover values, derived solely from the model’s predictions, were referred to as the ‘AI-alone’ values for each image. The model’s performance was evaluated by comparing its predictions against the expert-derived values for the same images (see later).

### AI + Citizen analysis platform

**Fig. 3:**
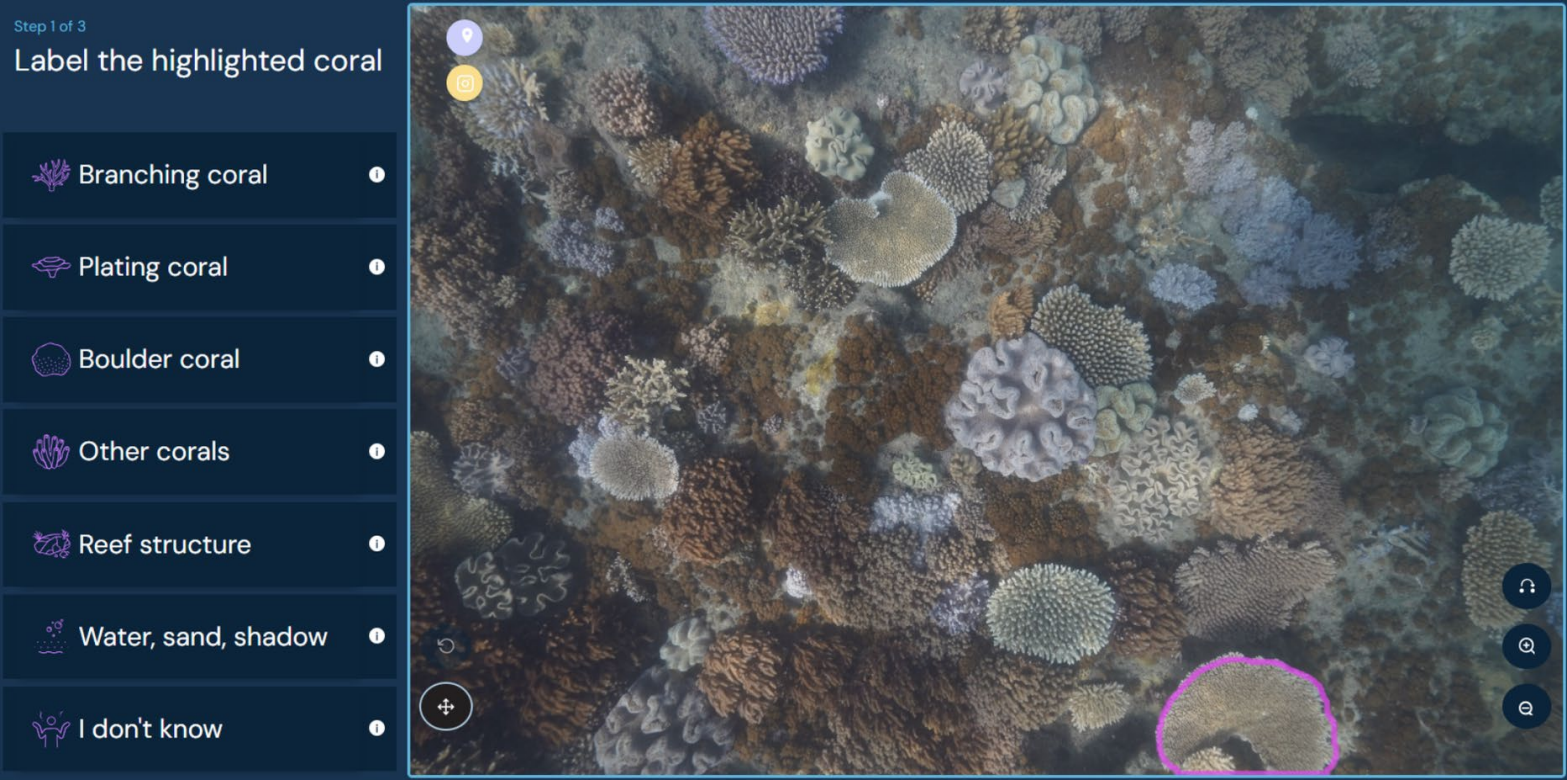
The Great Reef Census online analysis platform. Citizen scientists assigned labels to polygons generated by a segmentation model identifying distinct objects (the same segmentation model that was used in the AI-alone analysis). The highlighted polygon to label can be seen in the bottom right corner. Credit: greatreefcensus.org

An online platform was created (www.greatreefcensus.org/analysis) where citizen scientists assign labels to polygons for each image to derive coral cover of each category (Fig. 3). The polygons were generated by the segmentation model that was used in the AI-alone analysis. However, the AI + Citizens analysis differs from the AI-alone analysis because the polygons were labelled by citizen scientists. The label options for the AI + Citizens analysis were the same categories used for the expert and AI-alone analysis (Fig. 2). Platform users were primarily volunteers, including the public, school children, and corporate staff partners in Corporate Social Responsibility programs. A 3-min video was provided when users first logged in to the platform to explain how to identify each category, with a help page available at all times. Floating pop-ups on the platform were also available on the image analysis page to remind users how to identify each group if required. For each analysis, the cover of each category was calculated in the same method as the expert and AI-only analysis, i.e. coral cover as a percentage of colonisable area in the image. When multiple users analysed the same image, the average of all user results was used.

The platform randomly assigned images to users in real-time, prioritising images with the fewest analyses complete. For example, if some images had already been analysed by two other users, the platform would only present images to users that had been analysed once. All images with the lowest number of complete analyses were equally likely to be assigned to a user, so that the images from a site were analysed by several online users.

The online platform was operational for 11 months (April 2023–March 2024), during which 150,391 analyses of 20,879 images – each analysed multiple times – were completed by 6052 individual citizen scientists from 70 countries. Instructions on the online platform were available in English only.

### Data analysis

We conducted a series of tests to examine the effectiveness and reliability of the citizen science method for collecting coral cover data. Based on the accuracies of other common tools (Leujak & Ormond, 2007), we chose ± 5% absolute difference from expert values as an ecologically relevant accuracy target for broadscale reconnaissance; for example to be useful for distinguishing healthy from unhealthy reefs. To combine the relative strengths of the AI-alone and the AI + Citizens methods, the most accurate analysis method for each coral type was used in a ‘best’ method for all images. Next, while the mean of all images might be accurate, some management applications require coral cover estimates specifically at unhealthy reefs, in which case the method needs to be tested for images with low coral cover (0–20% coral cover). We disaggregated images into coral cover bins of 10% increments for each coral type as determined by the experts, so that results can be interpreted within a diversity of reef contexts. For example, citizen science may overestimate low coral cover images and underestimate at high coral cover: a common problem for bounded proportion (0–100) metrics (Ferrari & Cribari-Neto, 2004). Consequently, any such bias may systematically over- or underestimate coral cover at individual locations. Therefore, within each 10% bin with at least 80 images, we obtained the mean accuracy of images using our ‘best’ method for each coral cover category. Images were re-assigned to bins for each coral cover category. We then used simulations to determine how many images are needed to ensure a site estimate reliably falls within ± 5% accuracy. This is required because although the mean value of all images may be accurate, there is variability in the accuracy of coral cover derived from any one image. Greater variability in accuracy among images will require more images from each site to obtain a reliably accurate mean site value. Finally, we performed power analyses to determine how many images are needed from a site to detect a 10% difference in coral cover, with 80% power, of each coral category.

### Accuracy of coral categories per image

#### AI-alone

To determine the accuracy of the AI-alone method for each coral type, the mean expert result of each coral cover category $j$ for each image $i$ (${Expert}_{ij}$, % cover) was subtracted from the AI-alone result of the same image (${AI}_{ij}$, % cover) to obtain an absolute percent difference ${Accuracy}_{ij}^{AI}$(% cover):

1 $${Accuracy}_{ij}^{AI} ={AI}_{ij}- {Expert}_{ij}$$

This was repeated for all coral categories. For example, if the AI output for branching coral was 5% and the expert value was 10% for the same image, the AI-alone accuracy was described as − 5%, i.e. AI underestimated the expert value by 5%.

#### *AI* + *Citizen*

Similarly, to determine the accuracy of the AI + Citizen analysis for each image and coral category (${Accuracy}_{ij}^{Citizens}$, % cover), the mean expert result was subtracted from the mean AI + Citizen result to obtain an absolute percent difference:

2 $${Accuracy}_{ij}^{Citizens} ={Citizens}_{ij}- {Expert}_{ij}$$

#### ‘Best method’ accuracy

Given the relative strengths of the AI-alone and AI + Citizen results individually, we combined the results to achieve the ‘best’ method for analysing citizen science images. The best method used the more accurate – using the mean of all images – of the AI-alone or AI + Citizen method for each coral type (${Accuracy}_{ij}^{Best}$) and applied it to all images.

### Images required per site

#### *Accuracy: number of images needed to reach* ± *5% accuracy*

The earlier analyses provide the overall accuracy of the methodology in extracting coral cover from an image. However, given the variation in accuracy among images, we need to know how many images are needed for a site to meet a mean accuracy of ± 5%. We defined mean accuracy of a site as the difference in derived mean coral cover compared to the expert’s mean of the same images, i.e. the value that would be extracted from the tool for management purposes. To answer this question, we ran a series of simulations. For each simulation run, we randomly sampled $n$ images from the entire image library and determined the mean accuracy ($\overline{{Accuracy}_{nj}}$, % cover) of each coral type $j$ in those images:

3 $$\overline{{Accuracy}_{nj}}= \frac{1}{n}\sum_{i=1}^{n}{Accuracy}_{ij}^{Best}$$

We conducted 10,000 simulation runs for each value of $n$ from 1 to 120 and plotted each run’s value for $\overline{{Accuracy}_{nj}}$.

#### Effect of multiple citizen analyses per image

An advantage of the AI + Citizen analysis over AI-alone is that multiple citizen scientists can analyse the same image to obtain a mean result. The mean result from many individual analyses may be more accurate than having one citizen scientist analyse each image. As a result, if images are analysed by multiple citizen scientists, we may need fewer images to meet an accuracy of ± 5% reliably, which can reduce the in-water survey effort. We assumed ‘reliably’ meant that an accuracy of ± 5% is achieved in 95% of simulation runs. For the coral types for which AI + Citizen analysis was the most accurate, we determined the effect of increasing the number of analyses on the probability of a site being within ± 5% of expert analysis. To achieve this, we repeated the simulations described in "Accuracy: number of images needed to reach ± 5% accuracy" while varying the number of analyses per image ($m$) from 1 to 6. Analyses were sampled with replacement from each image. To obtain the mean accuracy of an image $i$ with varying citizen scientist analyses ($v$):

4 $$\overline{{Accuracy}_{ijm}}= \frac{1}{m}\sum_{v=1}^{m}{Accuracy}_{jv}^{Best}$$

where $\overline{{Accuracy}_{ijm}}$ is the mean accuracy of coral category $j$ for an image $i$ with $m$ number of citizen scientist analyses ($v$). We determined the mean accuracy across $n$ images, given $m$ citizen scientist analyses per image, by:

5 $$\overline{{Accuracy}_{njm}}= \frac{1}{n}\sum_{i=1}^{n}{\overline{Accuracy}}_{ijm}$$

For each image count ($n$ = 1 to 120) and analysis count ($m$ = 1 to 6), the percent of runs that had a mean accuracy within ± 5% was noted (out of the 10,000 runs for each combination of image count and analysis count). This provided the minimum number of images needed per site to meet an accuracy of ± 5% in 95% of runs, to test if the number of images needed is reduced with more analyses completed per image.

#### Power analysis: number of images needed to detect 10% difference in coral cover

Once the minimum number of images to meet accuracy requirements for the methodology has been determined, there remains the question of capturing heterogeneity of the reefscape. A series of power analyses were performed to determine how many images per site are needed to distinguish between sites with a 10% difference in coral cover.

Images analysed by all methods (AI-alone, AI + Citizen and experts) were grouped according to survey site. Each site was categorised into 10% coral cover bins (0–10%, 10–20%, etc.) for each coral type according to expert values. The standard deviation of coral cover values at each site was determined for each coral type using our ‘best’ method to capture the variability when using citizen science methodology. Then, within each coral type and coral cover bin, the mean standard deviation of coral cover at all sites was calculated.

The mean standard deviation of sites for each coral type and coral cover bin was used to conduct a power analysis, aimed at determining the minimum number of images needed per site to detect a 10% absolute difference in coral cover (effect size) with a power of 0.8 and an alpha level of 0.05. Any sites with fewer than 10 images analysed were discarded for this analysis. Images were not mixed across sites to ensure the realistic heterogeneity of the reefscape was captured.

All statistical analysis was performed in R (R Core Team, 2010) and the *tidyverse* collection of packages (Wickham et al., 2019). The power analyses were performed using the *pwr* package (Champely, 2020).

## Results

### Accuracy of coral categories per image

#### *Mean accuracy of AI-alone and AI* + *Citizens*

**Fig. 4:**
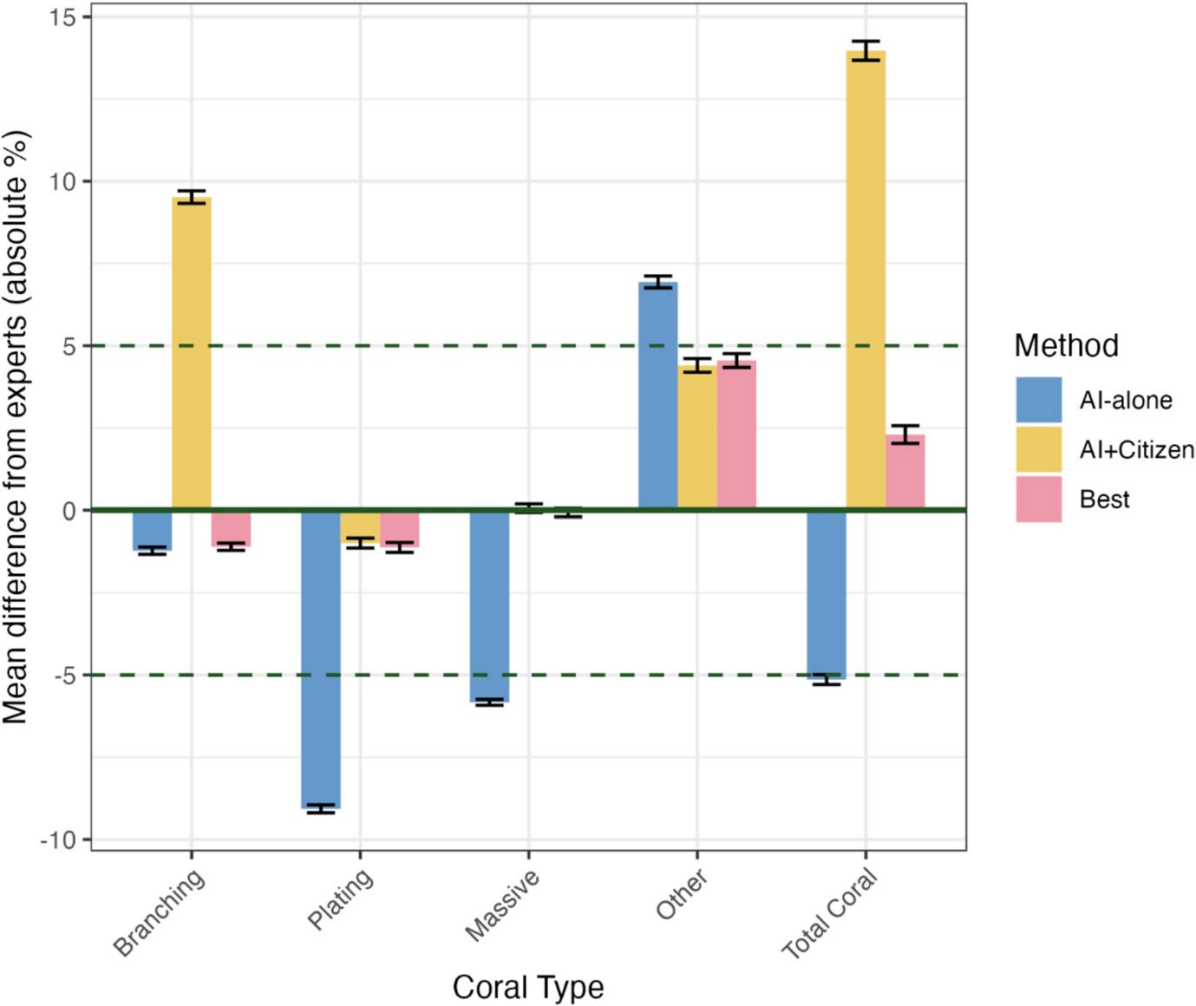
The mean accuracy of the AI-alone (7505 images), AI + Citizen (7790 images) and ‘Best’ (7608 images) method for each coral category. The *y*-axis is measured as the difference between the method’s output and the expert results for each same image. ‘Total Coral’ is the accuracy of the total benthic coral cover, i.e. the sum of the difference from expert analysis of all individual coral categories. ‘Other’ refers to all coral types except branching *Acropora*, plating *Acropora* and massive-form corals. Error bars show standard error of the mean. NB: Negligible differences are observed between the best method and the most accurate method for each coral type (e.g. AI-alone and best for branching coral) due to slight differences in which images were analysed for each method

The mean difference between the expert analysis and AI-alone analysis for all images (8086 images) ranged from − 9.1% for plating coral to + 6.9% for other coral (Fig. 4). The mean difference between expert analysis and AI + Citizen analysis for all images with at least 1 citizen analysis (7790 images) ranged from − 0.99% for plating coral to + 9.5% for branching coral (Fig. 4).

The AI-alone method was more accurate for branching coral cover, while the AI + Citizen method performed better for plating, massive and other coral cover. Therefore, the ‘best’ method combined AI-alone results for Branching coral with AI + Citizen results for the remaining coral types. The mean difference from experts using our best method was − 1.1% for branching coral, − 1.1% for plating coral, − 0.1% for massive coral and + 4.5% for other coral. Using our ‘best’ method, the mean difference from experts for total coral cover improved from − 5.1% (AI-alone) and + 13.9% (AI + Citizen) to + 2.3% (Fig. 4).

#### Disaggregating accuracy by reef state (coral cover)

**Fig. 5:**
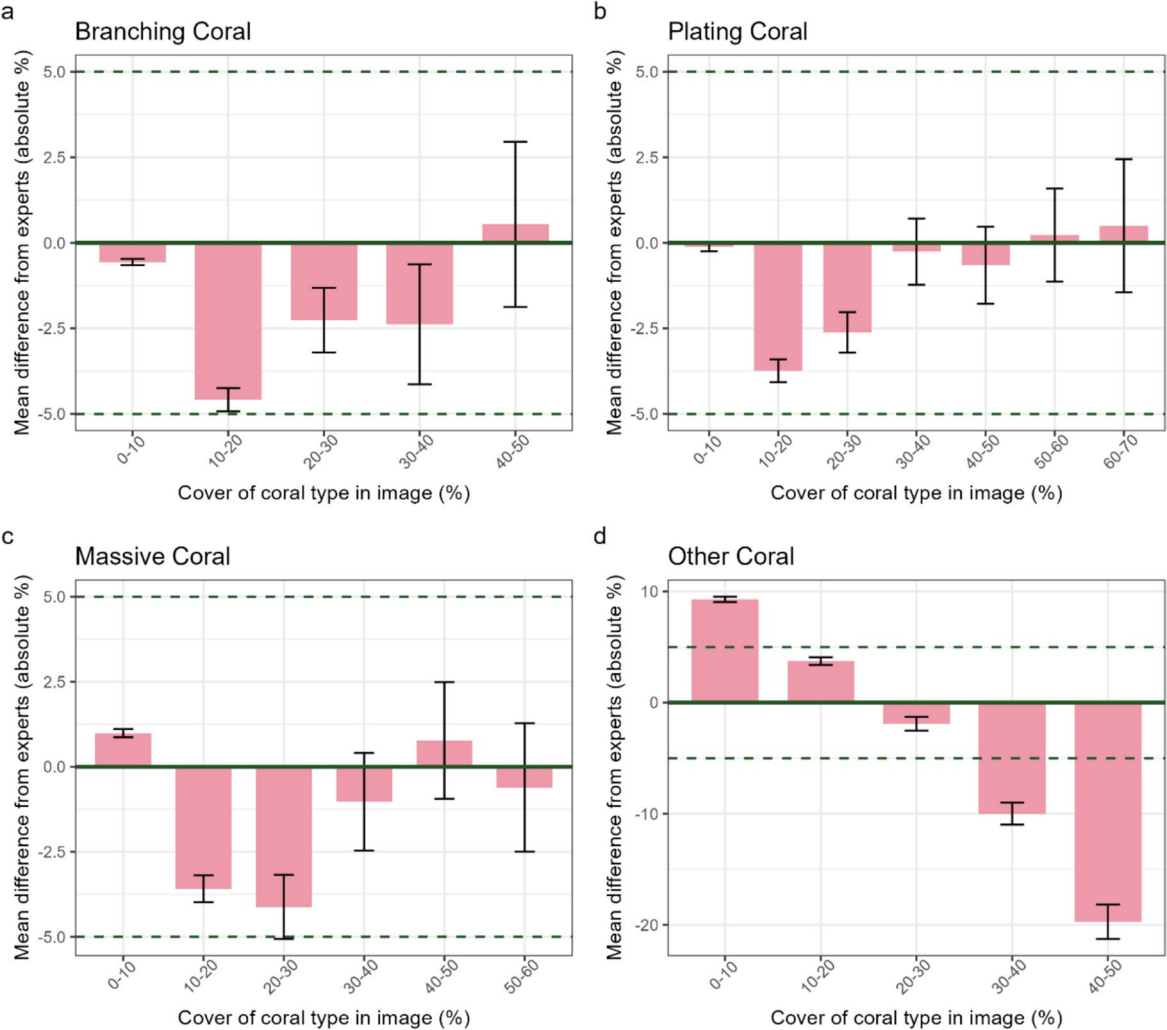
The mean accuracy of coral cover estimates for images from each 10% reef state bin using the ‘best’ method (minimum images per reef state bin = 80, because up to 80 images were needed to achieve accuracy in 95% of sites for all coral types with one citizen analysis complete; see Fig. 6, Plating coral panel). The *x*-axis represents the coral cover of the coral category according to expert analysis. Error bars show standard error of the mean; there were generally fewer images available at higher coral cover bins, resulting in larger standard errors. Dashed horizontal lines show our desired accuracy threshold (± 5%). Note that the *y*-axis range differs in **d**

For branching, plating and massive coral, all reef state bins were within our target of ± 5% accuracy (Fig. 5), but other coral had higher error for low and high reef state bins, ranging from + 9.3% for 0–10% coral cover to − 19.7% for 40–50% coral cover (Fig. 5). There was higher uncertainty in mean accuracy at high coral covers due to small sample sizes (Fig. 5).

### Images required per site

#### Accuracy: number of images needed to reach ± 5% accuracy

**Fig. 6:**
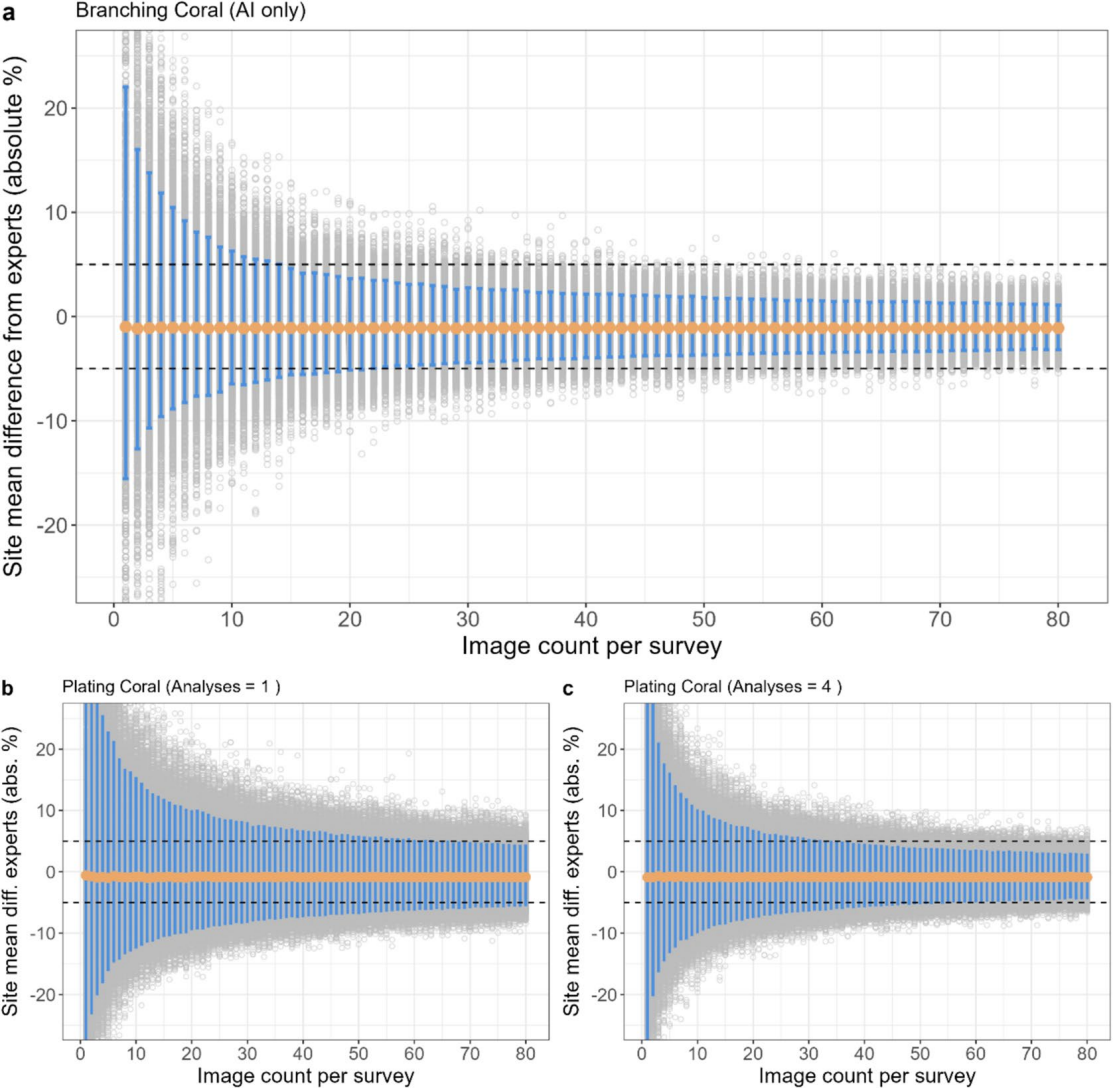

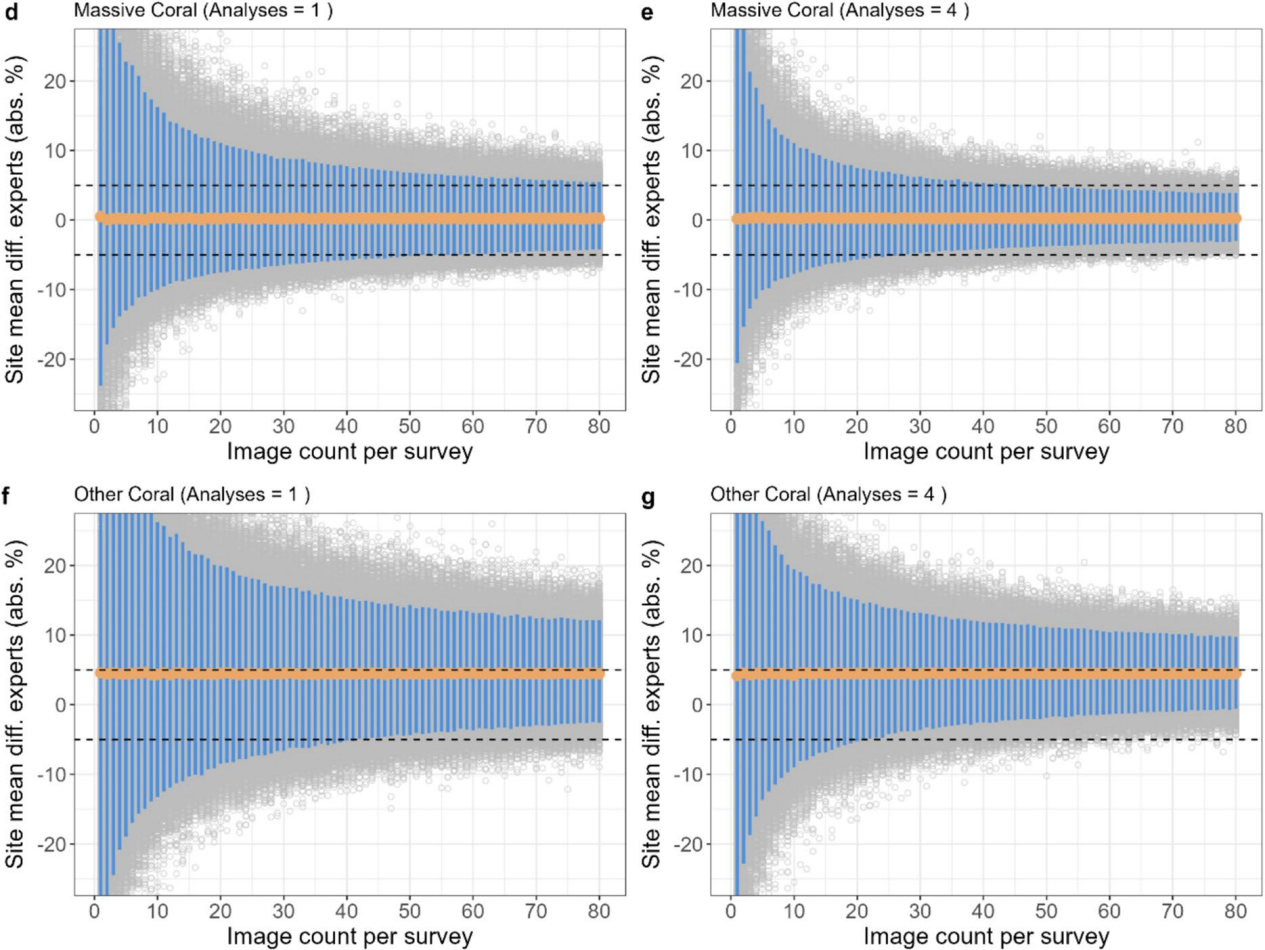
Mean site accuracy with increasing image count for branching coral using AI-alone (**a**). Plating coral with 1 citizen analysis per image (**b**). Plating coral with 4 citizen analyses per image (**c**). Massive coral with 1 citizen analysis per image (**d**). Massive coral with 4 citizen analysis per image (**e**). Other coral with 1 citizen analysis per image (**f**). Other coral with 4 citizen analysis per image (**g**). Each grey point represents the mean image accuracy of one simulation run of randomly sampled images (10,000 runs per image count value). The orange points represent the mean value of all simulation run means for each image count value. The blue bars show where 95% of simulation runs lie. The simulations were run up to 120 images per survey site, but the *x*-axis is truncated for clarity here

The simulations showed that increasing the number of images per site reduced the variability in mean site accuracy (Fig. 6). For example, with just one image per site, 95% of sites had differences from expert analysis ranging from − 11 to + 22% in absolute branching coral cover. In contrast, when 80 images were collected per site, 95% of sites showed differences within a narrower range of − 3 to + 1%. Consequently, collecting more images from each site increased the likelihood of the site accuracy meeting an accuracy of ± 5%. Branching coral – the only category in which AI-alone was most accurate – required 17 images per site for the mean accuracy to be within ± 5% accuracy for 95% of sites (Fig. 6a; Figure S1). Plating and massive coral needed less than 80 and 70 images, respectively, to achieve ± 5% accuracy for 95% of sites, but this varied depending on the number of citizen analyses completed on each image (see later). For other coral, as the number of images collected per site increased, the percent of sites that achieved ± 5% accuracy became asymptotic to about 60% (Fig. 6f, g).

#### Effect of multiple citizen analyses per image

For coral categories in which AI + Citizen was more accurate (plating, massive and other) than AI-alone, increasing the number of analyses per image reduced the number of images needed per site to achieve ± 5% accuracy, with diminishing returns (Fig. 6b–g). For example, with just one analysis per image, 80 (plating) and 70 (massive) images were needed to meet an accuracy of ± 5% for 95% of simulated sites, yet if 4 analyses were completed, then just 44 and 34 images, respectively, were needed (Fig. 6b–g; Figure S1). Completing 6 analyses per image only marginally reduced the required images to 40 and 31 images for plating and massive categories, respectively. In general, 4 analyses per image achieved high accuracy with efficient resource use, however this will vary depending on project goals and resource distribution across in-water survey and online analysis efforts.

#### Power analysis: number of images needed to detect 10% difference in coral cover

**Fig. 7:**
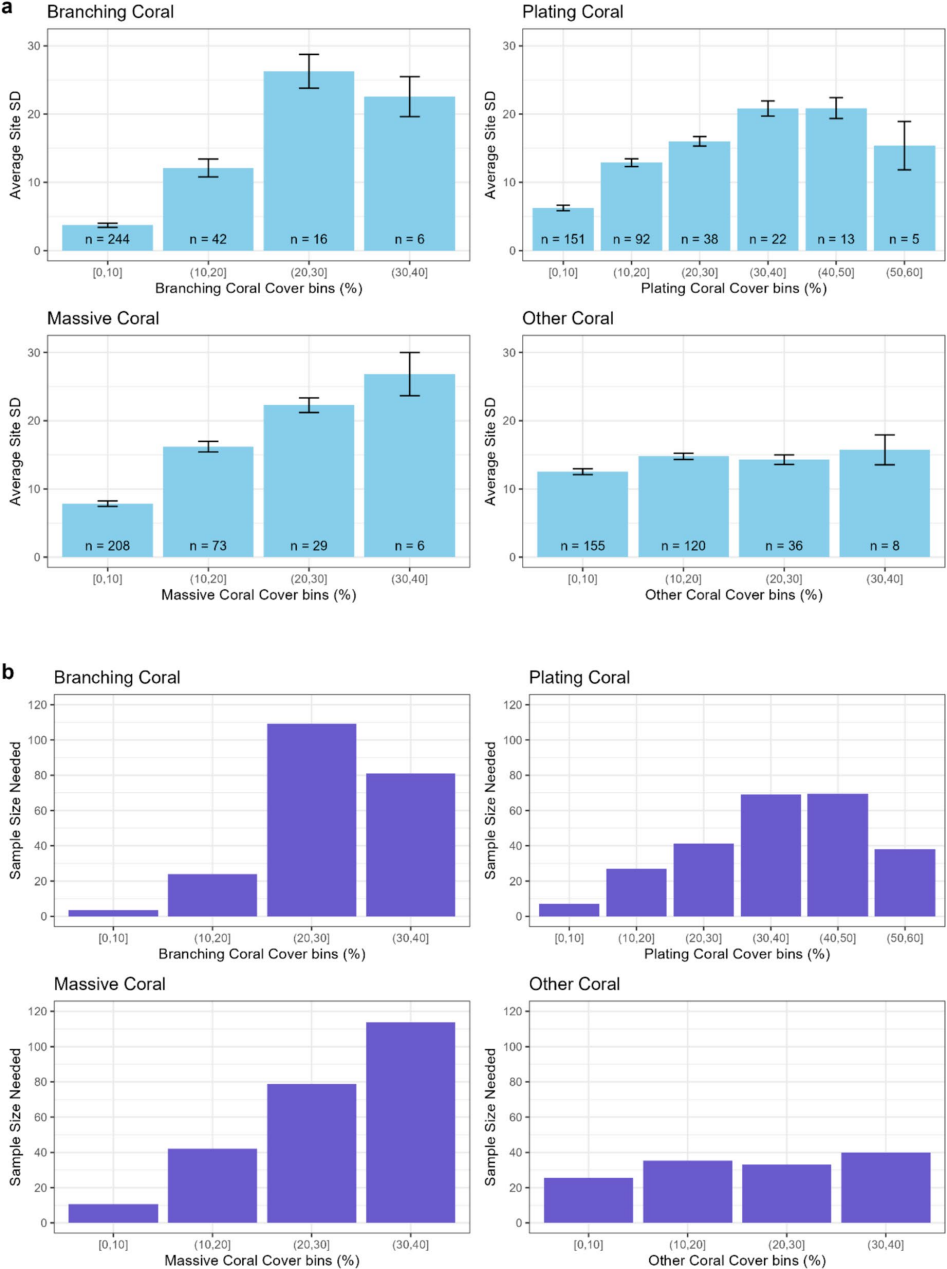
**a** Mean standard deviation of surveyed sites for each reef state bin and coral type. Error bars show standard error of the standard deviation. *n* Values inset show the number of sites in each column. **b** Power analysis results. Columns show the number of images required per site to detect a 10% difference in coral cover among sites based on their standard deviation (power = 0.8, alpha = 0.05)

The power analyses showed that the number of images required to detect a 10% difference in absolute coral cover ranged from 4 (branching coral 0–10%) to 114 per site (massive coral 30–40%; Fig. 7). Most of the tested categories required 80 images or less to detect a 10% difference in absolute coral cover of that category. Generally, more images were needed at higher coral covers. Few sites were available with coral cover greater than 50% in any coral category.

## Discussion

A combination of AI and non-expert human analysis of large field-of-view benthic images collected by citizen scientists can provide cover estimates of key coral categories that are accurate to within ± 5% of trained expert analysis. This accuracy was achieved at any level of coral cover for branching, plating and massive coral, but was only achieved for other coral in images with 10–30% cover. The level of citizen science effort required to meet ± 5% accuracy for the three key coral categories – up to 45 images per site analysed by four citizen scientists – is achievable based on previous participation in citizen science initiatives. Power analyses demonstrated that for some sites, more images are needed to detect a 10% change in coral cover and capture the heterogeneity of the reef than are necessary to be confident in the accuracy of the analysis method. Here, we discuss the practical application of these methods and considerations dependent on project goals.

### Varying the sampling protocol based on project goals

A project using a citizen science-based method similar to that presented here can adjust its sampling strategy based on the program goals and distribution of resources between in-water survey efforts and online citizen scientists (Table 2). If more resources are allocated to online citizen scientists than in-water sampling, the project could reduce the number of images collected, relying on increased citizen scientist analysis effort to maintain confidence in the results. Over the first 2 years of testing the online analysis platform, each image was analysed 5–6 times. The platform’s scalability suggests that this level of analysis can be sustained given that online analysis is cheaper and can be conducted globally, while in-water surveys require more resources and are restricted to local participants. Indeed, in some instances collecting fewer images per site and surveying more sites is a preferred approach, as more extensive online citizen science analysis could compensate for the lower image count.

**Table 2:** Example scenarios to illustrate the interplay between the number of images required to meet methodological accuracy levels and the number of images required to detect a 10% difference in coral cover between sites based on the results of the power analysis

Scenario	Required images relative to number needed for ± 5% methodological accuracy	Required images relative to number needed to detect a 10% difference
Low precision needed: 15–20% detectable difference in coral cover between sites	Similar	Fewer
High precision needed: 5% detectable difference in coral cover between sites	Similar	More
Approximate coral cover needed to distinguish very low and high coral cover reefs	Fewer	Fewer
Outplant restoration site with low, homogenous coral cover	Similar	Less than needed to meet ± 5% accuracy
Validate habitat maps of dominant coral type	Similar	Likely fewer

Fewer images will decrease the certainty in coral cover estimates, however this may be acceptable for some project goals. In most cases, the required number of images to meet desired methodological accuracy will need to be met, regardless of if the results of the power analysis need to be met. However, in some instances, a ± 5% accuracy target is higher than required and so fewer images can be collected to meet a lower accuracy target

For example, there is management interest in validating modelled habitat maps of key coral morphologies (Roelfsema et al., 2021). These maps predict the coral morphology most likely to dominate based on environmental factors such as wave energy and disturbance exposure. Such maps support research, ecological modelling and decision-making in management and restoration (Anthony et al., 2017; Bellwood et al., 2019; Pittman et al., 2007). However, the modelled predictions of dominant coral type often lack empirical validation. To validate these maps most effectively, it is essential to survey as many sites as possible given that dominant coral type can vary over short distances. Hence, using online citizen science analysis to improve accuracy and minimise image collection at any one site is preferred.

Sampling design can also be guided if the approximate condition of the reef is known a priori. For example, if a site is known to be heavily damaged with less than 20% coral cover, then the power to detect change is unlikely to be an issue if enough images are collected to meet accuracy needs for the sampling method (generally at least ~ 44 images with 4 analyses each). A similar approach may be taken to survey small scale restoration activity where most of the area can be surveyed directly and/or is likely to be highly homogenous (McLeod et al., 2022).

Similarly, if a project needs less accurate estimates of coral cover, say within ± 10%, fewer images are needed to be confident in the method. As coral cover increases, it is likely less important to obtain a highly accurate and precise estimate of coral cover. For example, a ± 10% range in possible values at 50% coral cover is unlikely to affect decision-making in the same way it would at 15% coral cover (Wickham et al., 2019), unless the goal is to track coral cover change precisely over time.

### Samples required compared to other tools

Here, we showed that useful broadscale reconnaissance survey data can be achieved with currently observed levels of citizen scientist engagement. In some situations, such as sites with branching 20–30% and massive coral 30–40%, more images were needed to detect a 10% difference in coral cover with sufficient power than were needed to be confident in the accuracy of the sampling method. The limiting factor at such sites may be the natural heterogeneity of the reef rather than the accuracy of the sampling method. This is reflected in traditional reef surveying methods such as photo quadrats and line transect point methods, which require sampling similar to or greater than needed here. For example, to detect a 20% relative difference in coral cover using photo quadrat methods, above 10% absolute cover, requires 38–48 (branching *Acropora*) and 111–141 images (massive *Porites*), or using line transect methods requires 990–15,450 (branching *Acropora*) and 820–8200 points (massive *Porites*) (Leujak & Ormond, 2007). Similarly, Carneiro et al. (2024) found that substantially more survey effort was required to achieve equivalent accuracy and precision by two common line transect survey methods, Reef Check and the Atlantic and Gulf Rapid Reef Assessment, compared to photo quadrats. To estimate coral cover with a 20% error margin, Reef Check required 1280–3080 line transect points and Atlantic and Gulf Rapid Reef Assessment required 1400–2200 line transect points (Carneiro et al., 2024).

The distribution of effort among the number of images collected per site, sites surveyed, and analyses completed per image will depend on the resource availability and goals of a program. However, the approximate requirements presented here are achievable based on experience. For example, while collecting 80 images per site (40 images each by two snorkellers), previous Great Reef Census expeditions with four participants have surveyed up to 124 sites across 42 reefs in 6 days (personal communication, A. Ridley, Citizens of the Reef). Similarly, in the first 2 years of the Great Reef Census operating, all images (up to 29,967 per year) have been analysed by at least 5 online citizen scientists with participants from 80 countries (unpublished data). Given this observed effort and the potential for widespread use by citizen scientists, such a method may expand data collection in resource-poor areas or provide an efficient complement to existing methods (Madin et al., 2019).

### Correcting for known inaccuracy

If there are systematic biases that cause known inaccuracies in a method, a correction offset can be included when reporting results (e.g. Eikelboom et al., 2019). For example, a 5% methodological overestimation may reduce the data’s reliability for management decision-making. Hence, any estimates of accuracy can be used as an offset to correct the data.

Here, applying a constant offset is likely suitable for branching, plating and massive coral estimates because all coral cover bins for these categories had similar accuracies that were reliably within ± 5% of the expert analysis. Applying such an offset should not affect the uncertainty of estimates, and therefore will not affect required sample size, because the offset is an absolute percentage of a proportion rather than a relative percentage offset (Eikelboom et al., 2019). However, care should be taken if applying an offset for Other coral results, which had more variable accuracy depending on coral cover level. Other coral was overestimated at low coral covers and underestimated at high coral covers, making it difficult to apply a constant offset. This may be a limitation of the current method, in that accurate estimates of cover can be provided for branching, plating and massive coral but total coral cover will be underestimated at sites with high other coral cover.

### Future improvements

The main drivers of improved performance in distributed data collection and analysis programs will likely be technological, although improved training of citizen scientists and program design can help. For example, anecdotally, we observed that poor-quality images appeared to be harder for both the AI and citizen scientists to analyse accurately. Poor quality images were commonly caused by human/camera error, poor water visibility or images captured more than 5 m from the reef. As camera technology improves and becomes cheaper, the occurrence of poor-quality images will likely reduce. Similarly, participants could be instructed to capture images closer to the sea floor, for example at 3 m instead of 5 m, especially in poor water visibility. Improved access to post-processing tools, such as automatic colour correction, can also improve image quality (Raveendran et al., 2021). Post-processing tools may also improve image segmentation performance in locations that consistently have higher turbidity, such as in-shore reefs with high agricultural or urban water run-off (Raveendran et al., 2021).

Major improvements may also be achieved by increasing the number of benthic categories that can be accurately measured. The other coral group here was the least accurate likely because it encompasses all coral types except our three key morphologies, making segmentation model training difficult (Rubbens et al., 2023). The uncertainty in other coral estimates may be resolved by disaggregating the category into distinct coral morphologies/taxonomies and through continual advances in deep learning (González-Rivero et al., 2020). This is likely feasible given the rapid progress in image segmentation using deep learning, with deep learning software already identifying some coral to the species level (González-Rivero et al., 2020).

In terms of training citizen scientists, clearer instructions for identifying dead coral may improve accuracy. Dead branching coral in particular – the only coral category for which AI-alone was more accurate than AI + Citizens – appeared to be poorly identified (personal communication, Citizens of the Reef). There is also evidence that in-water citizen scientists can be trained to detect more morphologies or groups of organisms than used here, including algae and sponges (Done et al., 2017). Indeed, more resource-intensive citizen science programs routinely assess dozens of benthic categories (Done et al., 2017). However, there is a trade-off between data quality and scalability; higher taxonomic resolution data currently requires high quality photographs or multi-day, in-water participant training that intrinsically limits the program’s potential span of data collection. Still, better online training and incorporating deep learning may enable online citizen scientists to identify more taxonomies, given their success in the present study.

Improving citizen science training in the tool and expanding the benthic categories that can be categorised, alongside general improvements in segmentation model technology, may enable such a tool to be used in other reef habitats not considered here. We have not tested the methodology across different reef habitats, for example Pacific vs. Atlantic reefs or tropical vs subtropical reefs. However, given the success seen here and clear paths for improvement, it is likely that new models can be trained for new use cases and environments, including reef systems dominated by non-*Acropora* species.

While we only assessed the cover of live coral, a similar approach could be applied to measures of changing coral condition. For example, given the increasing rate of marine heatwaves and global coral bleaching events (Hughes et al., 2018), there is interest in collecting in-water data across broad spatial scales on the rate and impact of coral bleaching. While broadscale data on bleaching can be collected from aerial surveys, in-water data is usually required to prevent false negatives. In other words, the presence of bleaching can be seen from the air but the absence of bleaching needs in-water data to confirm if there was live coral present (Hedley et al., 2016). Similarly, in-water data is used to measure the spread and impact of pest species or corallivory, such as the crown-of-thorns starfish on the Great Barrier Reef (Matthews et al., 2024). Deep learning models are already being developed to detect coral bleaching, corallivory and disease (Clampitt, 2023; Wong et al., 2024) and their inclusion in a tool such as we describe here may expand their application.

## Conclusions

A program such as the Great Reef Census demonstrates how technology, particularly deep learning, can lower the barrier to entry for citizen science, allowing non-experts to contribute to accurate coral reef data collection. This approach can enable large-scale participation globally. While not a replacement for more detailed scientific monitoring, the method may provide a complementary tool that can support coral reef management, especially in resource-limited regions, by offering an accessible and cost-effective method for broadscale surveying of key coral morphologies.

## Supplementary Information

Below is the link to the electronic supplementary material.

Supplementary file1 (DOCX 634 KB)

## Data Availability

The datasets generated during the current study are available from the corresponding author on reasonable request.
