# Supplementary material for: Broadscale reconnaissance of coral reefs from citizen science and deep learning
Source: Environ Monit Assess. 2025 Jun 27;197(7):814. doi: 10.1007/s10661-025-14261-6 (PMC12202624; doi:10.1007/s10661-025-14261-6)
Supplement: Supplementary file 1 — Supplementary file1 (DOCX 634 KB) [file 10661_2025_14261_MOESM1_ESM.docx]

**Broadscale reconnaissance of coral reefs from citizen science and deep learning**

Christopher L. Lawson1, Kathryn M. Chartrand2, Chris M. Roelfsema3, Aruna Kolluru4, Peter J. Mumby1

1 Marine Spatial Ecology Laboratory, Centre for Conservation and Biodiversity Science, School of Environment, University of Queensland, Brisbane, Australia

2 Centre for Tropical Water and Aquatic Ecosystem Research, James Cook University, Cairns, Australia

3 Marine Ecosystem Monitoring Lab, School of Environment, The University of Queensland, Brisbane, Australia

4 Dell Technologies Inc., Round Rock, USA

Corresponding author:

Christopher L. Lawson

Christopher.lawson@uq.edu.au

**Supplementary material**


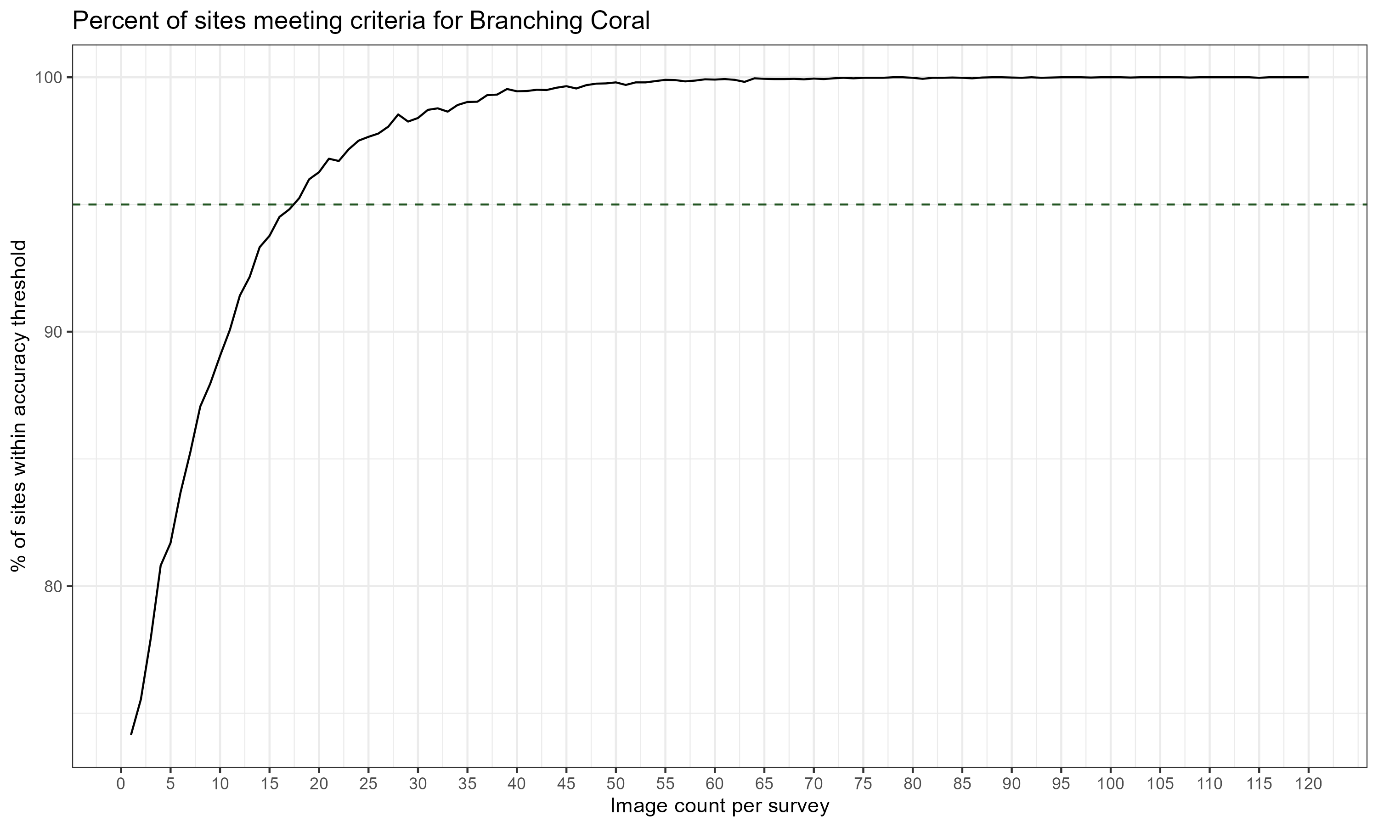


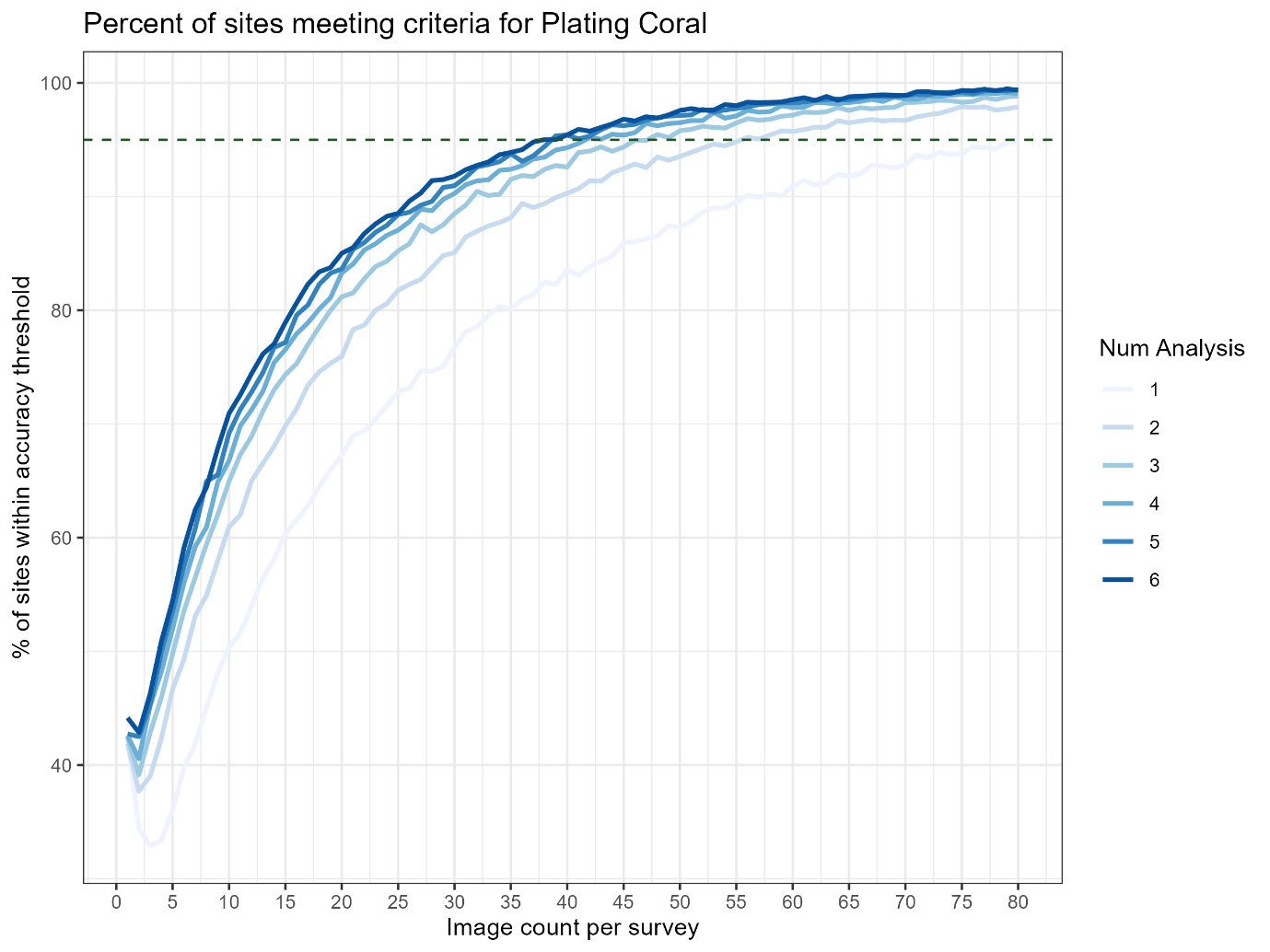


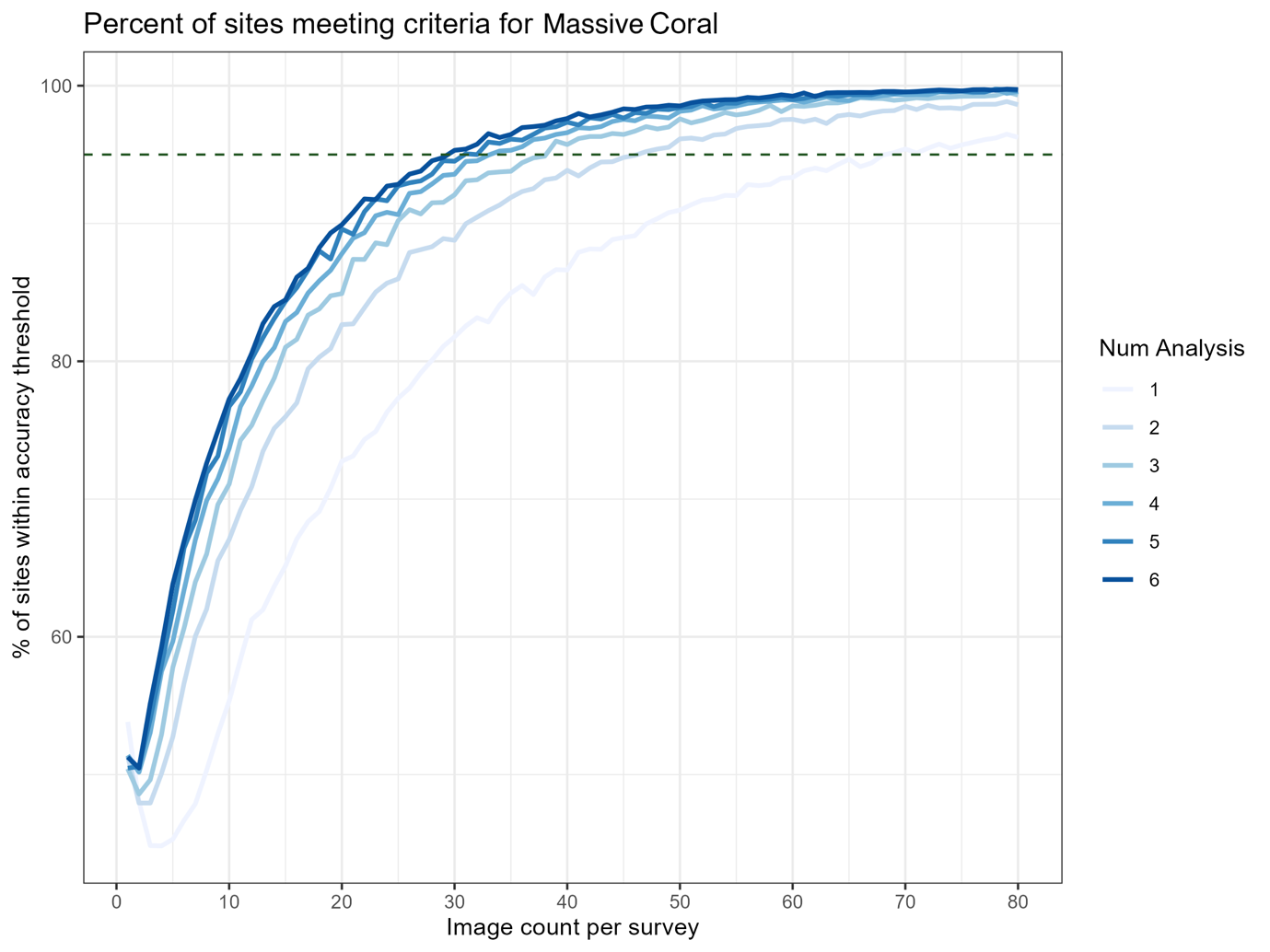


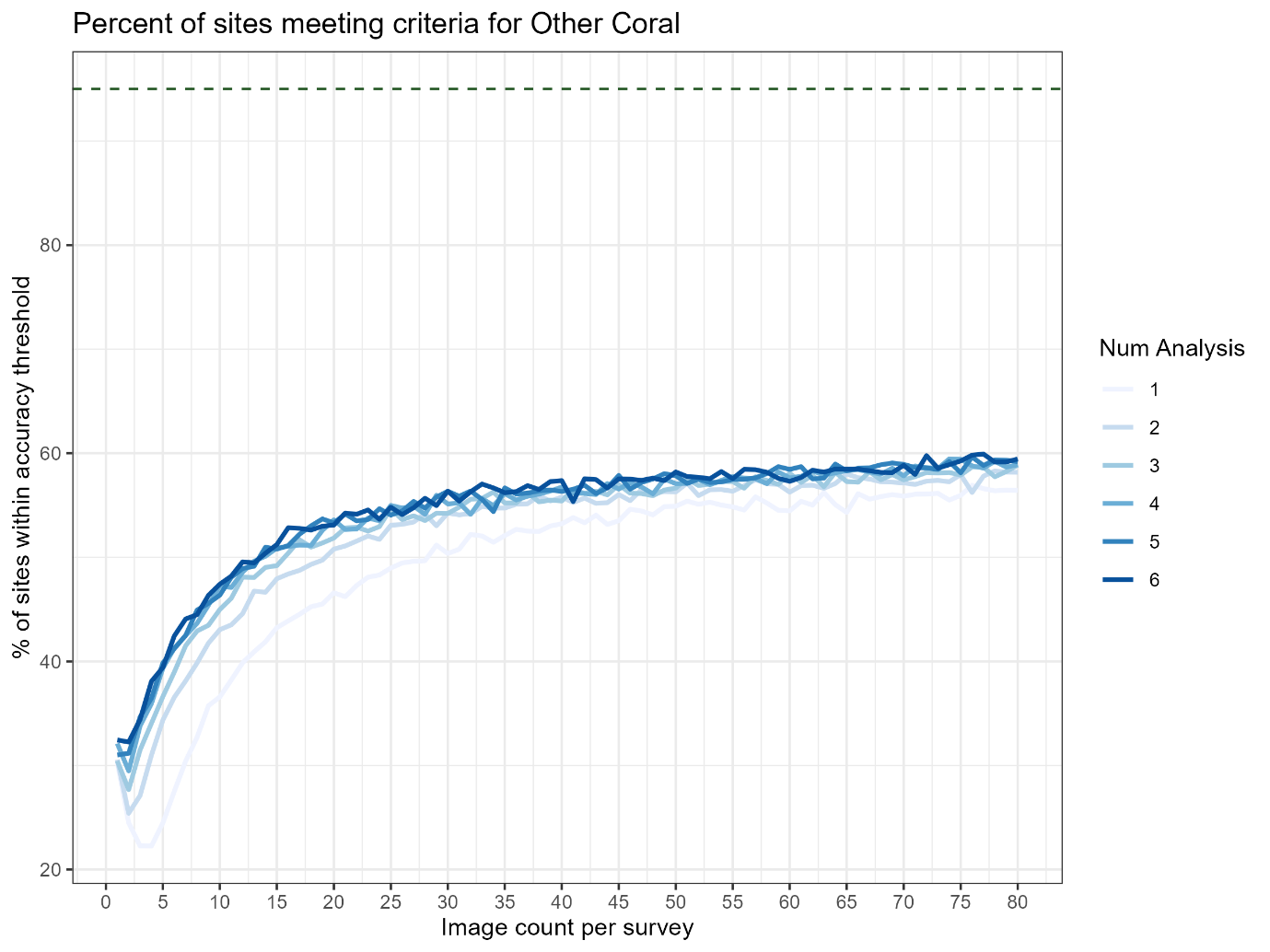


**Fig. S1** Cumulative percent of simulated sites that meet ±5% mean accuracy relative to expert analysis. Branching coral shows the results of AI-alone. The remaining categories show results from the AI+Citizen online analysis, with each line representing the results of varying the number of citizen analyses performed on each image. The dashed horizontal line shows 95% of simulated sites
